# Correction to “Self‐collected vaginal specimens for human papillomavirus testing and guidance on screening exit: An update to the American Cancer Society cervical cancer screening guideline”

**DOI:** 10.3322/caac.70066

**Published:** 2026-01-28

Perkins RB, Wolf AMD, Church TR, et al. Self‐collected vaginal specimens for human papillomavirus testing and guidance on screening exit: An update to the American Cancer Society cervical cancer screening guideline. *CA Cancer J Clin*. 2026;e70041. doi:10.3322/caac.70041

In this article by Perkins, et al., there is an incorrect statement in Table 2 at column 3, row 4.

The original text read:

“Does not qualify for screening exit; very limited data.”

The statement should read:

“May qualify for screening exit; very limited data.”

In Table 2, the footnote was incorrectly stated. The correct footnote should read:

“Abbreviations: ACOG, American College of Obstetricians and Gynecologists; AIS, adenocarcinoma in situ; ASCCP, American Society of Colposcopy and Cervical Pathology; CIN, cervical intraepithelial neoplasia; CIN2, cervical intraepithelial neoplasia grade 2; CIN3, cervical intraepithelial neoplasia grade 3; HIV, human immunodeficiency virus; HPV, human papillomavirus; HSIL, high‐grade squamous intraepithelial lesion; LSIL, low‐grade squamous intraepithelial lesion.”

In Table 3, the footnote was incorrectly stated. The correct footnote should read:

“Abbreviations: CIN3+, cervical intraepithelial neoplasia grade 3 or worse; FDA, US Food and Drug Administration; HPV, human papillomavirus.”

On page 3 first column, “or the Abbott Alinity m with the Evalyn Brush or Qvintip swab (part of simpli‐COLLECT kit)” should not be italicized.

We apologize for these errors.

